# An Urgent Need to Restrict Access to Pesticides Based on Human Lethality

**DOI:** 10.1371/journal.pmed.1000358

**Published:** 2010-10-26

**Authors:** Matthew Miller, Kavi Bhalla

**Affiliations:** 1 Department of Health Policy and Management, Harvard School of Public Health, Boston, Massachusetts, United States of America; 2 Department of Global Health and Population, Harvard School of Public Health, Cambridge, Massachusetts, United States of America

## Abstract

Matthew Miller and Kavi Bhalla discuss new research findings from Andrew Dawson and colleagues on the human toxicity of pesticides in Sri Lanka, and call for urgent reclassification of agricultural pesticides to help reduce suicides by poisonings.

**Linked Research Article**

Dawson AH, Eddleston M, Senarathna L, Mohamed F, Gawarammana I, et al. (2010) Acute human lethal toxicity of agricultural pesticides: a prospective cohort study. PLoS Med 7(10): e1000357. doi:10.1371/journal.pmed.1000357

In a prospective cohort study of patients presenting with pesticide self-poisoning, Andrew Dawson and colleagues investigate the relative human toxicity of agricultural pesticides and contrast it with WHO toxicity classifications, which are based on rat toxicity.

Agricultural pesticides account for at least 250,000 suicide deaths each year, making pesticides the single most common means of suicide worldwide [1],[2]. The proportion of suicide deaths attributable to pesticide self-poisoning varies considerably across the world: in Europe and the Americas fewer than 5% of suicide deaths involve pesticides; in the Eastern Mediterranean, African, and Southeast Asian regions approximately 20%–25% involve pesticides; and in the Western Pacific region more than half of all suicides are pesticide related [1]. In aggregate, pesticide poisoning is involved in one-third of all suicides. In Sri Lanka, the site of the study by Andrew Dawson and colleagues published in this week's *PLoS Medicine* [3], more than half of all suicide deaths in 2005 were due to pesticides [4].

## Means Restriction: An Evidence-Based Approach to Preventing Suicide

Over the past two decades, a series of targeted legislative initiatives in Sri Lanka culminated in the withdrawal of World Health Organization (WHO) class I pesticides and, eventually, endosulfan, resulting in a fall in the incidence of suicide by 50%. This decline in suicide by pesticides occurred without a compensatory increase in suicide by other methods [5]. Other notable examples of large-scale population level reductions in suicide incidence due to decreased access to and/or availability of highly lethal and commonly used suicide methods (an approach known as means restriction) have occurred in Samoa and the United Kingdom. In Western Samoa the rise and fall of suicides, but not suicide attempts, closely tracked the introduction and later banning of paraquat on the island [6]. In the UK during the 1960s, a shift in the source of household heating away from coal-gas (capable of producing lethal levels of carbon monoxide) to detoxified natural gas was followed by a 30% decline in national suicide rates. As in Sri Lanka, the dramatic decline in overall suicide incidence in the UK was driven by a decline in method-specific suicides (i.e., in carbon monoxide suicides) without compensatory increases in suicide by other (i.e., non-carbon monoxide related) methods. This natural experiment in the UK is known as the “Coal Gas Story” [7],[8], and is perhaps the most famous example of means restriction at the population level.

These examples, along with ecologic and case control studies in the United States that found the availability of and access to household firearms heightens the risk of suicide for all members of the household [9], support the claim that means restriction is one of only two approaches to suicide prevention with a strong evidence base [10]—and the only approach for which such large-scale reductions in suicide incidence have been observed at the population level.

## A New Study of Pesticide Lethality

The study on pesticide lethality by Dawson and colleagues [3] adds an important observation to the evidence base for pesticide restrictions in South Asia. In the current study, data from two referral hospitals in Sri Lanka were used to calculate formulation-specific case fatality ratios for several pesticides used in acts of intentional self-harm. The authors found that the human toxicity of many pesticides commonly used in Sri Lanka today is sharply discordant with current WHO toxicity rankings (which are based predominantly on oral LD50 in rat models). Dawson and colleagues not only identify several pesticides commonly used in suicide attempts that are far more likely to prove lethal to humans than is suggested by current WHO toxicity classification (e.g., paraquat), they also identify readily available and significantly less toxic alternative formulations (e.g., glyphosate) with similar agricultural indications.

Dawson and colleagues thoughtfully address the chief data limitations of their study. First, they note that although case fatality rates from a hospital study cannot account for deaths that occur outside of the medical system, over 90% of known pesticide poisoning deaths in Sri Lanka occur in medical facilities. Second, they point out that despite the fact that their data do not account for pesticide self-ingestion if the ingestion did not result in a hospital visit, there are no systematic differences in pesticide formulations causing death in the referral hospital compared with pre-referral locations (primary hospitals and home). Consequently, the very large differences in the *relative* case fatality across pesticides observed in the current study are not likely to be biased by this limitation.

## Policy Implications: An Urgent Need to Modify WHO Classifications

Findings from the current study by Dawson and colleagues suggest persuasively that human toxicity data should be incorporated into regulatory decisions. Integrating human toxicity data into the WHO toxicity schema has the potential not only to further reduce suicide rates in Sri Lanka, but also to dramatically reduce suicide incidence throughout the developing world—where hundreds of thousands of lives hang in the balance. As such, the WHO should immediately reclassify some formulations (e.g., dimethoate, fenthion, and paraquat) since the lethality of these compounds is several-fold higher than alternatives within the same chemical and functional class. For many other pesticides, more precise measures of human lethality are needed, highlighting the importance of sustainable funding streams to develop and maintain surveillance systems that collect data on intentional self-harm and for an iterative process whereby pesticides are periodically reclassified as new data become available.

Considered in light of growing evidence that some pesticides can be eliminated without adverse effects on agricultural output or production costs, organizations in a position to influence governmental action should use data from Dawson and colleagues and from the original Sri Lankan success story with class I pesticides to urge countries to restrict access to pesticides that are commonly used and lethally toxic to humans. In addition, national and international funding agencies that want to maximize their impact on the global burden of suicide should devote additional resources to research in means restriction. Indeed, it would be interesting to know what proportion of suicide prevention funding is devoted to means restriction—and why, as we suspect, the amount is disproportionately small by any sensible measure of the toll of preventable death and the promise of this often neglected, yet profoundly effective, approach.

## Conclusion

Findings from the current study by Dawson and colleagues have helped refine human toxicity estimates for pesticides in use today. Better surveillance data and additional research will, eventually, lead to additional refinements. In the meantime, while we wait for these refinements, we must not ignore what, thanks to Dawson and colleagues, we already know. As Bradford Hill noted nearly half a century ago [11], the fact that all scientific knowledge is provisional does not, in his memorable words, confer upon us a freedom to ignore the knowledge we already have, or to postpone the action that it appears to demand at a given time.
